# The hidden burden of sleep in adolescents with idiopathic generalized epilepsy

**DOI:** 10.1002/epd2.70288

**Published:** 2026-07-11

**Authors:** Carlo Alberto Quaranta, Martina Paola Zanaboni, Elena Ripamonti, Gaia Garghetti, Massimiliano Celario, Serena Grumi, Helene Vitali, Valentina De Giorgis, Ludovica Pasca

**Affiliations:** ^1^ Department of Brain and Behaviour Neuroscience University of Pavia Pavia Italy; ^2^ Child Neurology and Psychiatry Unit IRCCS Mondino Foundation, Full Member of European Reference Network EpiCARE Pavia Italy; ^3^ Developmental Psychobiology Lab IRCCS Mondino Foundation Pavia Italy; ^4^ Unit for Visually Impaired People Istituto Italiano di Tecnologia Genoa Italy

**Keywords:** adolescents, emotional–behavioral symptoms, executive function EFs, idiopathic generalized epilepsy IGE, sleep quality

## Abstract

**Background:**

Idiopathic Generalized Epilepsies (IGEs) are electroclinical syndromes characterized by distinct seizure types, EEG patterns, and presumed polygenic inheritance. While seizure prognosis is often favorable, sleep disturbances, behavioral and emotional difficulties, and executive function impairment are common, especially during adolescence.

**Objective:**

To investigate the relationship between subjective sleep quality, emotional–behavioral functioning, and executive functions in adolescents with IGE.

**Methods:**

In this single‐center observational study, 28 adolescents (mean age 15.0 ± 2.4 years) with IGE were evaluated with the following standardized measures: PSQI (sleep), BRIEF‐2 (executive functions), CBCL and YSR (emotional–behavioral profile), SAFA‐A/‐D/‐S (internalizing and somatic symptoms). Dream‐related experiences were also collected. Pearson correlations tested behavioral and emotional associations with sleep quality.

**Results:**

Poor sleep quality (PSQI ≥5) occurred in 28.6% of patients, although only 14.8% reported sleep problems in their clinical history. Poor sleepers showed significantly higher anxiety, depression, somatic complaints, and stress‐related symptoms (*p* < .05). Executive function impairment, particularly in emotional control and cognitive flexibility, was more frequent in this group.

**Significance:**

Sleep quality is an under‐recognized but clinically relevant dimension in patients with IGE, closely linked to emotional vulnerability and executive function difficulties. Routine sleep screening and targeted sleep‐focused management should be integrated into pediatric IGE care to support psychological well‐being.

**Key points**

1. Poor sleep is frequent but under‐recognized in adolescents with idiopathic generalized epilepsy (IGE). In our study cohort, 28.6% of subjects had a reduced sleep quality.
2. Worse sleep quality correlates with executive function impairment, particularly in cognitive flexibility and emotional regulation.
3. Poor sleepers show higher emotional–behavioral symptom burden, including anxiety, depression, somatic complaints, and stress‐related problems across multiple informants.
4. Integrating routine sleep assessment and targeted sleep management might improve overall neuropsychological outcomes in pediatric IGE.

## 1 INTRODUCTION

Although formally subsumed under the umbrella of Genetic Generalized Epilepsies (GGEs) in the 2017 ILAE classification, the Idiopathic Generalized Epilepsies (IGEs) remain a clinically meaningful entity due to their distinctive features.^1^, ^2^, ^3^ Traditionally, including four core syndromes, Childhood Absence Epilepsy (CAE), Juvenile Absence Epilepsy (JAE), Juvenile Myoclonic Epilepsy (JME), and Generalized Tonic–Clonic Seizures Alone (GTCS), IGEs account for 15%–20% of all epilepsies, with typical onset between childhood and early adulthood.^4^ Patients generally show a favorable response to antiseizure medications (ASMs), yet up to one quarter of patients remain drug‐resistant.^5^

IGEs do not appear to significantly impair global cognitive functioning, which generally remains within the normal range; however, growing evidence suggests the presence of subtle deficits in executive functions (EFs) and learning abilities, negatively affecting social functioning.^6^ Psychiatric comorbidities—particularly mood and anxiety disorders as well as impulsivity and certain personality traits—along with adverse psychosocial outcomes, have been considered not merely consequences of a chronic disease but integral components of the IGE endophenotype.^7^, ^8^, ^9^

Within this complex clinical landscape, sleep has emerged as a critical yet under‐recognized factor influencing disease expression. The epilepsy–sleep relationship is bidirectional: on one side, interictal epileptic discharges and ASMs can lead to impaired sleep quality, indeed IGEs typically present an increased spike–wave activity during sleep and transition to sleep^1^; on the other side, sleep fragmentation and deprivation lower seizure threshold and trigger seizures and interictal epileptic activity.^10^, ^11^, ^12^ Polysomnographic studies in adults with IGEs consistently demonstrate reduced sleep efficiency, increased arousals, and abnormal distribution of slow‐wave and REM sleep, changes possibly linked to cognitive dysfunction.^11^, ^13^, ^14^, ^15^, ^16^ A recent meta‐analysis further highlights lower sleep efficiency and total sleep time, as well as prolonged REM latency, in IGEs compared to controls.^17^ Pediatric data, though more limited, indicate similar alterations with associated attentional and behavioral disturbances.^14^, ^15^

Beyond seizures, sleep disruption impacts learning and memory processes, as well as mood and self‐regulation.^18^ In clinical practice, the systematic assessment of sleep quality and the promotion of sleep hygiene in epileptic patients are strongly recommended,^19^ not only to optimize seizure control, but also as integral components of overall health care. A deeper understanding of the interplay between sleep quality, cognition, and emotional–behavioral symptoms in IGEs is crucial to guide individualized management.

The present study investigates sleep quality, cognitive performance, EFs, and emotional–behavioral profiles in a pediatric IGE cohort, with the aim of disentangling their interrelationships to inform targeted diagnostic and therapeutic strategies.

## 2 MATERIALS AND METHODS

### 2.1 Study design

This was a single‐center observational study primarily aimed at evaluating sleep quality in adolescents with IGEs. Secondary objectives were to explore the associations between sleep quality, cognitive functioning, EFs, and emotional‐behavioral profiles in this population.

The study was approved by the Ethics Committees of the IRCCS Mondino Foundation (Comitato Etico della Lombardia 6, Approval Number: 2023‐3.11/169) and registered at ClinicalTrials.gov Identifier: NCT06782074. Participants were consecutively recruited from outpatient clinics during routine clinical activities. All eligible patients who agreed to participate and provided written informed consent were included. Data collection began in May 2023 and ended in October 2024.

### 2.2 Participants

Adolescents (11–18 years) with IGE, diagnosed according to ILAE criteria by a trained epileptologist, were recruited.

Inclusion criteria were (1) a diagnosis of IGE, (2) age between 11 and 18 years, and (3) adequate compliance with study procedures.

Exclusion criteria were (1) a diagnosis of structural or cryptogenic epilepsy or any epilepsy other than IGE; (2) age below 11 or above 18 years old; (3) visual or motor impairments of such severity as to invalidate test administration; (4) a history of significant neurological or psychiatric conditions (e.g., brain malformations, stroke, head injury, schizophrenia, etc.); (5) intellectual disability (ID); and (6) inadequate compliance with study procedures.

#### 2.2.1 Demographic and clinical data

Demographic and clinical data were collected at the time of enrollment through direct interviews and review of medical records. The collected information included sex, age at seizure onset, age at last seizure, time from seizure onset to evaluation, epileptic syndrome diagnosis, seizure type and frequency, seizure freedom, concomitant ASMs, sleep disorders, and family history of epilepsy or psychiatric disorders.

### 2.3 Measures and procedures

#### 2.3.1 Sleep quality

All participants underwent subjective sleep quality assessment using the Pittsburgh Sleep Quality Index (PSQI), a validated self‐report questionnaire that evaluates sleep quality and disturbances across seven components: subjective sleep quality, sleep latency, sleep duration, habitual sleep efficiency, sleep disturbances, use of sleep medication, and daytime dysfunction.^20^

To explore dream activity and experience, three self‐report instruments were used: the Dream Recall Frequency Scale, the Van Dream Anxiety Scale, and the Manheim Dream Questionnaire (MADRE).^21^, ^22^

#### 2.3.2 Cognition and executive function

Cognitive functioning was assessed using the Full‐Scale Intelligence Quotient (FSIQ) obtained from either the Wechsler Intelligence Scale for Children–Fourth Edition (WISC‐IV)^23^ or the Wechsler Adult Intelligence Scale–Fourth Edition (WAIS‐IV)^24^ according to patient's age.

Furthermore, each participant completed the Behavior Rating Inventory of Executive Function–Self Report Version (BRIEF–SR),^25^ for executive functioning assessment. Caregivers also completed the parent version of the BRIEF‐2. The BRIEF‐2^25^ includes 7 scales: (1) Inhibition as ability to control impulses and stop inappropriate behavior; (2) Shift as cognitive flexibility and ability to transition between tasks; (3) Emotional Control as regulation of emotional responses; (4) Initiation as ability to begin tasks and generate ideas; (5) Working Memory as holding and manipulating information in mind; (6) Planning/Organization as managing complex tasks and setting goals; and (7) Monitoring as tracking performance and self‐correction. In addition, there are 4 Indices: Behavioral Regulation Index (BRI) includes Inhibition and Shift; Emotional Regulation Index (ERI) includes Shift and Emotional Control; Cognitive Regulation Index (CRI) includes Initiation, Working Memory, Planning/Organization, Monitoring; Global Executive Composite (GEC) as an overall measure of executive functioning.

#### 2.3.3 Behavior, emotion, and affectivity

To assess behavior and mental health, parents completed the Child Behavior Checklist (CBCL/6–18),^26^, ^27^ a standardized questionnaire widely used to identify behavioral/emotional problems and assess social competencies in children and adolescents. The CBCL consists of two main sections: one addressing social competence/adaptive functioning, and the other focusing on behavior problems. Based on responses, scores are computed for eight syndrome scales: Anxious/Depressed, Withdrawn/Depressed, Somatic Complaints, Social Problems, Thought Problems, Attention Problems, Rule‐Breaking Behavior, and Aggressive Behavior. These are further aggregated into three broadband scales: Internalizing Problems, Externalizing Problems, and Total Problems. In parallel, participants also completed the Youth Self‐Report (YSR), providing a self‐reported measure of emotional and behavioral functioning.

To dive deeper into anxiety and depression symptoms, the Psychiatric Scales for Self‐Administration for Youths and Adolescents (SAFA)^28^ were used. This psychometric tool assesses a wide range of psychological symptoms and conditions in children and adolescents; it consists of six self‐report scales that can be administered either individually or collectively. For the purposes of this study, we used the following three SAFA subscales: SAFA‐A (Anxiety), SAFA‐D (Depression), and SAFA‐S (Somatic Symptoms and Hypochondria).

### 2.4 Statistical approach

Descriptive statistics were computed to characterize the sample in terms of demographic (gender and age at testing) and epilepsy‐related variables. For continuous variables, means and standard deviations were calculated.

Pearson's correlation coefficients were used to assess the relationship between epilepsy‐related variables, emotional profile, cognitive profile, and subjective sleep quality.

The association between sleep quality (assessed with PSQI) and executive functioning (BRIEF scores) and emotional–behavioral problems (CBCL, YSR, SAFA scores) was examined using two approaches: (i) Pearson's correlation analysis, treating the PSQI global score as a continuous variable, significance levels were adjusted for multiple comparisons using the false discovery rate (FDR) procedure (Benjamini–Hochberg, *q* = .05); (ii) participants were divided into two groups based on their PSQI total score (0–30), following established clinical criteria: a score of ≤5 indicated good sleep quality (group 1), while a score >5 indicated poor sleep quality (group 2). This dichotomization allowed for a clearer distinction between participants with good versus poor sleep, providing a more homogeneous and clinically meaningful representation of the sample. At this stage, independent samples *t*‐tests were conducted, comparing participants with poor sleep quality (PSQI >5) and good sleep quality (PSQI ≤5) on all other outcome measures.

## 3 RESULTS

### 3.1 Demographic and clinical data

Our sample cohort was composed of 28 patients (11 males, 39.3%; 17 females, 60.7%) with a diagnosis of IGE.

Demographic and epilepsy‐related characteristics are presented in Table 1.

**TABLE 1:** Demographic and clinical data (including epilepsy‐related variables) of our cohort.

Demographic and clinical variables	IGE patients (*N* = 28)
Age (years)	Mean = 15.0 (SD 2.4)
Sex	Males: 11 (39.3%) Females: 17 (60.7%)
Age at seizure onset (years)	Mean = 10.53 (SD = 4.48)
Age at last seizure (years)	Mean = 13.28 (SD = 3.61)
Time from seizure onset to evaluation (years)	Mean = 4.06 (SD = 3.62)
Time from last seizure to evaluation (years)	Mean = 1.70 (SD = 1.84) Median = 1.08 Range: 1–71 (months)
Epilepsy diagnosis	CAE: 7 (25%) JAE: 8 (28.6%) JME: 6 (21.4%) GTCA: 7 (25%)
Current seizure frequency	Seizure‐free: 21 (75%) 1–3/year: 3 (10,7%) 4–9/year: 4 (14,3%)
Concomitant antiseizure medications	*N* = 28 Mean = 1.32 (SD .75)
Antiseizure medication distribution	Valproic Acid (VPA): 14 Levetiracetam (LEV): 9 Ethosuximide (ETS): 5 Lamotrigine (LMT): 4 Clobazam (CLB): 4 Topiramate (TPM): 1
Self‐reported sleep problems	*N* = 5 (16.1%)
Concomitant psychological support	Supportive psychological counseling = 5 (16.1%) Structured psychotherapy = 2 (6.45%)
Family history of epilepsy	*N* = 9 (29%)
Family history of psychiatric disorders	*N* = 5 (16%)

### 3.2 Sleep

Sleep features were assessed using the PSQI questionnaire. A total PSQI score above the value of 5 was found in 8 patients (28.6%). Sleep latency greater than 16 min was reported by 16 patients (57.1%), and a sleep duration of less than 7 h was observed in 8 patients (28.6%). Detailed results are shown in Table 2 and Figure 1.

**TABLE 2:** Dichotomized PSQI assessments of our cohort and PSQI total score.

Sleep variable	*M* (min–max)	SD
Subjective sleep quality	.82 (0–3)	.905
Sleep latency (general)	.89 (0–3)	.956
Sleep duration	.5 (0–3)	.923
Latency in past month	.32 (0–3)	.723
Nocturnal/early morning awakenings	.96 (0–3)	.693
Awakenings due to urination		
Breathing problems		
Snoring		
Awakenings due to cold		
Awakenings due to heat		
Negative dreams		
Pain		
Use of sleep medication	.07 (0–2)	.378
Daytime dysfunction	.79 (0–3)	.917
Total PSQI score	4.36 (0–15)	3.592

Abbreviations: max, maximum; min, minimum; PSQI, Pittsburgh Sleep Quality Index; SD, standard deviation.

**FIGURE 1:**
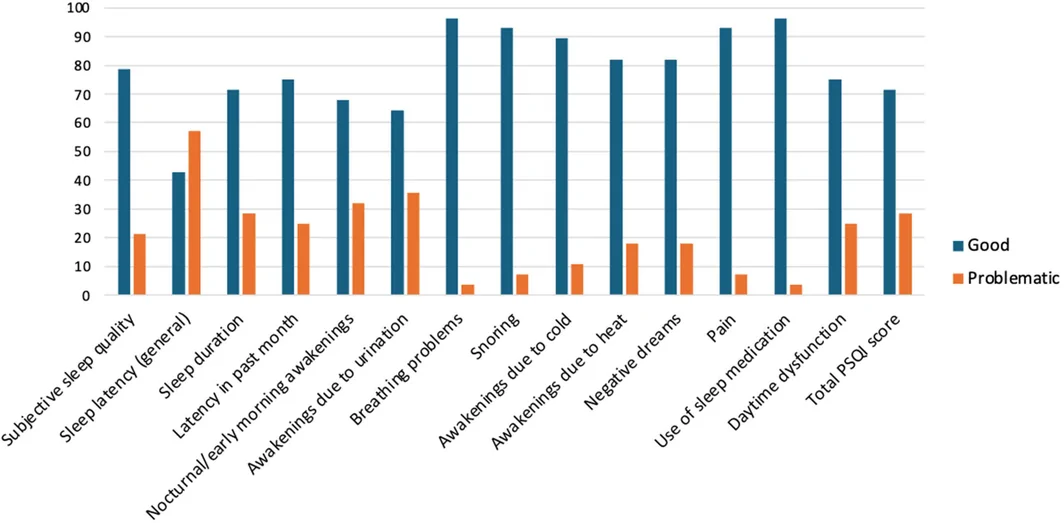
Dichotomized PSQI scoring among sleep subscales, highlighting the presence/absence of sleep issues.

### 3.3 Dream activity

Dream activity questionnaires (explorative analyses) were completed by 25 patients, of whom 14 (56%) reported habitually remembering their dreams. Most patients did not report issues related to nightmares, while approximately half (48%) expressed an interest in dreams. In depth, at least 20% of patients highlighted sleep difficulty due to nightmares.

Full details are reported in the Supplementary results section.

### 3.4 Neuropsychological data

#### 3.4.1 Cognitive profile

Regarding cognitive functioning, our sample performed within an average range FSIQ (mean 99.83, SD 11.3; minimum 84 – maximum 132). Subscales scores were as follows: Verbal Comprehension Index (VCI; mean 105, SD 12.05), Perceptual Reasoning Index (PRI; mean = 103.78, SD = 11.45), Working Memory Index (WMI; mean = 89.74, SD = 13.03), and Processing Speed Index (PSI; mean = 95.13, SD = 13.46).

#### 3.4.2 Executive functions

EFs were assessed using both the parent and self‐report versions of the BRIEF‐2. According to parent reports, the most frequently affected domains included Working Memory (15.4%), Task Monitoring (19.2%), and Planning/Organization (19.2%). Self‐reports also indicated difficulties, particularly in Emotional Regulation (14.3%), Shift (10.7%), Self‐Monitoring (10.7%), and Working Memory. Full details are reported in the Supplementary results section.

#### 3.4.3 Emotional and behavioral features

Emotional and behavioral functioning was assessed using the CBCL, YSR, and SAFA‐A, ‐D, and ‐S.

Among the clinically relevant domains of CBCL, the highest frequencies of clinical scores were observed for internalizing problems (20%), somatic complaints (19.2%), anxiety problems (26.9%), depressive problems (16%), and stress‐related problems (16%).

As far as YSR, the most affected domains included anxious/depressed (19.2%), stress‐related problems (19.2%), internalizing problems (15.4%), and withdrawn/depressed (15.4%).

At SAFA, the most affected domains were feelings of inadequacy and low self‐esteem (21.4%), generalized anxiety (17.9%), depression (14.3%), and simulation (14.3%). Somatic symptom scales were only mildly affected, with scores above the cut‐off in 3.6% of patients.

Full details are reported in the Supplementary results section.

### 3.5 Correlation analyses

#### 3.5.1 PSQI total score and neuropsychological variables

Table 3 highlights significant findings from the correlation matrix between the total PSQI score and data of executive functioning and emotional–behavioral assessments in self‐reports. No significant associations were found between PSQI scores and CBCL scores.

**TABLE 3:** Correlation analyses between the PSQI total score and emotional, behavioral, and EF assessment.

	PSQI correlation coefficient (*R* pearson)	*p*‐value (*significance FDR)
*BRIEF self‐report*		
Self‐monitoring	.491**	.008*
Shift	.620**	.000*
Emotion regulation	.573**	.001*
Task completion	.582**	.001*
Working memory	.560*	.002*
Planning/organization	.490*	.008*
BRI	.399*	.036*
ERI	.657**	.000*
CRI	.578**	.001*
GEC	.609**	.001*
*SAFA*		
Anxiety	.533**	.003*
Generalized anxiety	.477*	.010*
Social anxiety	.428*	.023*
School‐related anxiety	.512**	.005*
Depression	.452*	.016*
Feelings of inadequacy, low self‐esteem	.469*	.012*
Despair	.469**	.012*
Simulation	−.481**	.010*
Somatic symptoms and hypochondria	.842**	.000*
Somatic symptoms	.832**	.000*
Hypochondria	.588**	.001*
*YSR*		
Internalizing problems	.667**	.000*
Externalizing problems	.529**	.005*
Total problems	.689**	.000*
Anxious/depressed	.636**	.000*
Somatic complaints	.737**	.000*
Social problems	.483*	.014*
Thought problems	.663**	.000*
Attention problems	.541**	.005*
Aggressive behavior	.567**	.003*
Depressive problems	.690**	.000*
Anxiety problems	.573**	.002*
Somatic problems	.585**	.002*
Oppositional problems	.710**	.000*
Obsessive‐compulsive problems (ASEBA)	.535**	.005*
Stress problems (ASEBA)	.636**	.000*

Abbreviations: BRIEF, Behavior Rating Inventory of Executive Function; SAFA, Self‐administered Psychiatric Scales for children and adolescents; YSR, Youth Self‐Report.

* *p* < .05;

** *p* < .01.

#### 3.5.2 Dichotomized PSQI and clinically related variables

Significant differences (*p* < .05) from the *t*‐test between the two PSQI groups and clinical‐related variables were found.

Individuals with higher PSQI scores, indicating worse sleep quality, showed a significantly older age at last seizure (mean ± SD = 16.09 ± 2.08 years) compared to those with better sleep quality (mean ± SD = 12.43 ± 3.13 years). A trend toward a later age at seizure onset was also observed in the poorer sleep quality group (13.21 ± 2.31 years vs. 10.01 ± 4.83 years; *p* = .072). No significant difference emerged for the number of ASMs taken at the time of evaluation.

#### 3.5.3 Dichotomized PSQI and executive functions in self‐reports

Significant differences (*p* < .05) in self‐reported executive function assessments between PSQI groups were observed in Shift, Emotion Regulation, ERI, CRI, and GEC, as illustrated in Figure 2. Participants with poorer sleep quality showed greater impairments in emotional control (*t*(26) = 2.49, *p* = .019), cognitive flexibility (Shift: *t*(26) = 2.35, *p* = .027), and both cognitive (*t*(26) = 2.12, *p* = .044) and emotional regulation indexes (*t*(26) = 2.72, *p* = .012), as well as in the Global Executive Composite (*t*(26) = 2.36, *p* = .026). Overall, poorer sleep quality was associated with broader executive functions impairment, encompassing deficits in both cognitive control and emotional self‐regulation.

**FIGURE 2:**
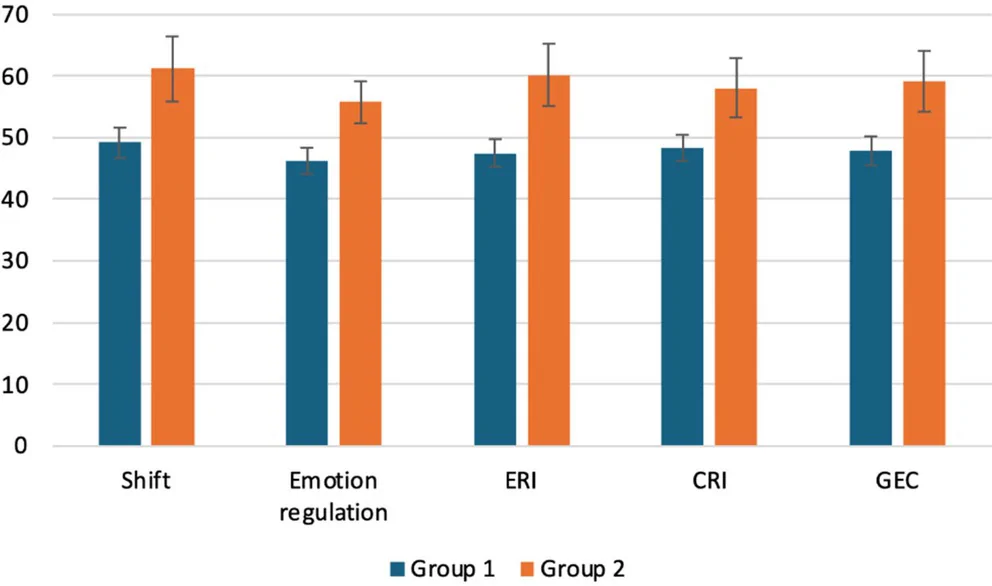
Correlation analyses between dichotomized PSQI and EF self‐reported assessments. CRI, Cognitive Regulation Index; DF, degree of freedom; ERI, Emotional Regulation Index; GEC, Global Executive Composite; SD, standard deviation; *t*, *t*‐test.

#### 3.5.4 Dichotomized PSQI and affectivity

Significant differences (*p* < .05) were observed between PSQI groups in patient‐questionnaires (SAFA‐A, SAFA‐D, SAFA‐S, YSR) and parent‐CBCL. These results are shown in Table 4 and represented in Figure 3.

**TABLE 4:** *T*‐test between dichotomized PSQI (group 1 = PSQI score <5; group 2 = PSQI score >5) and SAFA and CBCL assessments.

Variable	Group 1 mean (SD)	Group 2 mean (SD)	*t*	DF	*p* (two‐tailed)
*SAFA*					
Somatic symptoms & hypochondria	43.74 (5.67)	56.67 (10.85)	3364	10.12	.007
Somatic symptoms	44.37 (5.80)	57.00 (12.04)	2987	9.81	.014
Hypochondria	42.84 (3.83)	53.89 (11.97)	2703	8.79	.025
*CBCL*					
Oppositional behavior	52.76 (3.78)	56.78 (4.27)	2471	24	.021
*YSR*					
Internalizing problems	52.82 (10.16)	63.67 (12.88)	2360	24	.027
Total problems	50.88 (8.31)	61 (11.93)	2538	24	.018
Anxious/depressed	56.76 (7.30)	66.11 (10.91)	2614	24	.015
Thought problems	53.35 (3.99)	61.38 (10.82)	2736	23	.012
Depressive problems	54.00 (5.82)	65.11 (9.53)	3707	24	.001
Oppositional problem	52.41 (2.69)	58.25 (6.86)	2324	23	.005
Obsessive‐compulsive ASEBA	55.82 (7.63)	63.11 (9.12)	2167	24	.04
Stress problems ASEBA	55.76 (7.52)	65.67 (10.94)	2726	24	.012

Abbreviations: DF, degree of freedom; SD, standard deviation; *t*, *t*‐test.

**FIGURE 3:**
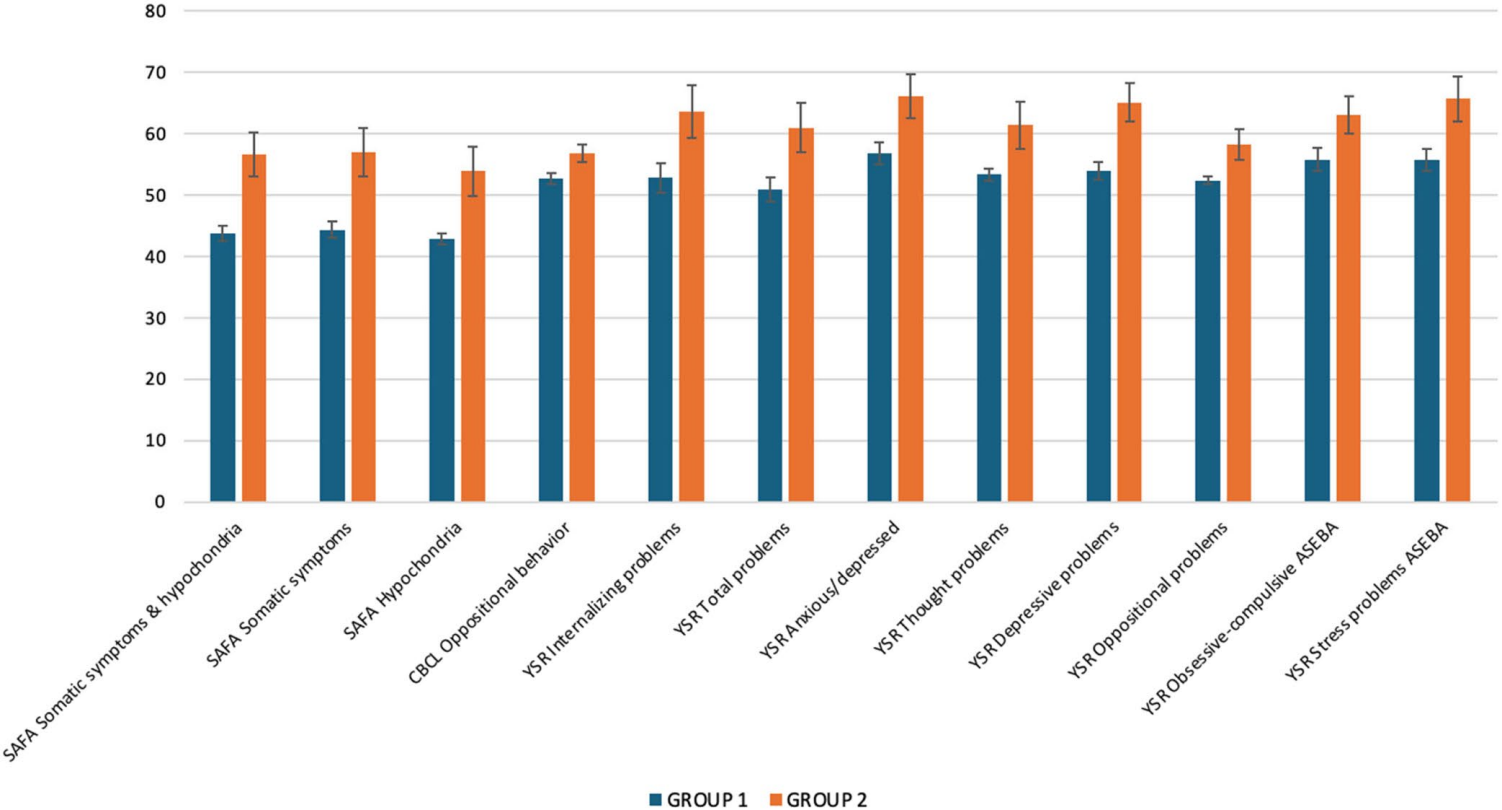
Correlation analyses between dichotomized PSQI and Emotional and Behavior assessments (SAFA and CBCL‐YSR). CBCL, Child Behavior Checklist; SAFA, Self‐administered Psychiatric Scales for children and adolescents; YSR, Youth Self‐Report.

Poor sleepers (PSQI ≥5) report significantly higher levels of somatic complaints, oppositional behavior, anxiety, depression, and stress‐related symptoms. These affective and behavioral difficulties were consistently confirmed by both self‐reports and parental assessments.

## 4 DISCUSSION

To our knowledge, this is the first study to jointly assess sleep quality, emotional–behavioral symptoms, and executive functions within a single IGE pediatric cohort. While previous work has typically addressed these domains mainly in adult patients or in isolation, our integrated approach highlights that poor sleep quality may underlie broader behavioral and emotional difficulties in IGE.

Sleep disturbance emerged as a relevant clinical issue. Although only 14.8% of participants spontaneously reported sleep problems, standardized PSQI scores revealed poor sleep quality in 28.6%, with frequent complaints of prolonged latency, reduced sleep time, nocturnal awakenings, and daytime dysfunction. These results align with previous evidence on sleep quality measured by the PSQI in IGE adults^17^ and in part mirror the physiological sleep changes typical of adolescence, a developmental phase particularly vulnerable to sleep–wake dysregulation.^18^ The well‐known bidirectional link between sleep and epilepsy reinforces this susceptibility: seizures and ASMs disrupt sleep, whereas sleep loss facilitates epileptiform activity.^11^, ^29^, ^30^, ^31^ Subjects with a more recent seizure occurrence and a later age at epilepsy onset were more likely to exhibit impaired sleep quality; this finding is consistent with previous evidence showing a higher subjective disease burden close to diagnosis and in cases of uncontrolled seizures, particularly in patients with drug‐resistant forms of IGEs.^13^

Poor sleep was significantly associated with lower EF performance according to self‐report BRIEF scores, showing lower scores in working memory and emotional control. Given that sleep disruption adversely affects daytime behavior and cognition, this link may reflect shared neurodevelopmental mechanisms. Frontal slow‐wave activity (SWA) undergoes major reorganization during adolescence, paralleling prefrontal cerebral maturation,^32^ and its disruption may mechanistically contribute to EF deficits.^33^ Consistent with meta‐analytic findings,^34^ IGE patients generally perform worse than controls in verbal, attentional, and memory domains, independent of seizure control or medication.^34^, ^35^, ^36^, ^37^ These impairments, attributed to fronto‐thalamo‐cortical dysconnectivity,^7^ may contribute to inefficient cognitive control. EF alteration also relates to behavioral problems frequently observed in pediatric epilepsy,^38^ as reduced inhibitory and monitoring capacities compromise social cognition and adaptive behavior.^39^, ^40^

Specific EF processes have been associated with emotional disorders, and it has been suggested that depressive mood and anxiety are linked to difficulties in maintaining task goals and that EF impairments contribute to the maintenance of these emotional disorders.^41^ Emotional regulation difficulties, such as blunted conflict responses, reduced empathy, and impaired recognition of negative emotions, likely reflect fronto‐limbic dysregulation and increase vulnerability to internalizing symptoms.^42^ Our findings confirm a substantial emotional burden, particularly anxiety and depression symptoms, in agreement with previous pediatric IGE reports^9^; Varesio et al.,^43^ for example, found anxiety symptoms in 40% and depression symptoms in 22.5% of adolescents with IGE using CBCL, YSR, and SAFA measures, rates closely matching those observed in our cohort. These symptoms often manifest through somatic complaints (e.g., fatigue, irritability, sleep problems), delaying recognition and appropriate referral. Sleep plays a crucial role in emotional regulation,^44^ and greater sleep difficulties may exacerbate challenges in affective control and emotion recognition, further amplifying anxiety and depressive symptoms in youth with IGEs. Therefore, routine emotional and sleep screening should be integral to epilepsy care.

Dream‐related findings further illustrate the subjective impact of sleep disturbance: 20% of patients highlighted sleep difficulty due to nightmares and mood alterations related to dream content, with an overall high dream recall. Although the neurobiology of dreaming in epilepsy remains incompletely understood, frequent nocturnal awakenings, common in our cohort, likely facilitate dream recall and enhance emotional salience,^45^ thereby compounding daytime distress and contributing to the broader sleep–emotion–cognition triad. In the absence of objective PSG data, speculations regarding REM‐specific mechanisms should be drawn with caution.

The finding that significant associations emerged between sleep quality and self‐reported measures of behavior and emotions, but not parent proxy reports, may reflect differences in the ability to recognize and report emotional and cognitive distress but can also suggest that sleep disturbances are more closely linked to subjective experiences of emotional and cognitive distress that subjects may be able to perceive and report better than caregivers. This interpretation may reflect informant‐specific effects that clinicians should be aware of and account for to achieve a comprehensive assessment.^46^

The limitations of this study include the lack of an objective sleep assessment and the absence of a report on EEG features; nevertheless, to date, IGE epileptiform activity has not been found to be associated with the behavioral phenotype.^47^

Although the PSQI is a well‐validated and widely adopted instrument, self‐report inherently captures the perceived experience of sleep rather than its objective features. This gap between subjective perception and objective sleep outcome represents an important caveat when interpreting the observed sleep–cognition–emotion associations and underscores the need for future studies incorporating polysomnography, actigraphy, or EEG spectral analyses to provide a neurophysiological basis driving these relationships and behavioral outcomes in pediatric IGEs.

Similarly, the absence of EEG data, including interictal discharge density and spike burden, could limit the results, and their contribution to the observed sleep–cognition–emotion associations cannot be excluded.

Furthermore, utilized scores were derived from patients' self‐reported psychiatric validated screening tools and not psychiatric diagnoses; as the chosen screening tools were validated and widely applied, a more detailed psychiatric assessment would probably not have affected the overall outcome. A subset of patients was receiving psychological support at the time of evaluation. Formal psychiatric comorbidities were excluded per protocol; however, a subset of patients was receiving psychological support, which in this context does not necessarily reflect underlying psychopathology. Given the small size of this subgroup, no formal statistical comparison of sleep quality between supported and non‐supported patients was performed. Future studies should incorporate standardized psychological interventions as a covariate, and longitudinal designs are warranted to explore the potential preventive role of psychotherapy in this population.

The cross‐sectional design precludes causal inference regarding the directionality of the observed associations. Finally, the study's sample size constitutes an additional limitation, reducing statistical power and limiting the generalizability of the findings. Replication in larger, multicenter, and syndrome‐stratified cohorts is required to extend these results. Our cohort comprised four distinct IGE syndromes pooled together, although this reflects that the shared genetic and neurophysiological substrate of the IGE spectrum, syndrome‐specific differences in sleep, including seizure timing distribution and semeiology, could not be explored given the insufficient sample size per subgroup. Syndrome‐stratified analyses in future larger cohorts are warranted. Although no significant difference in the number of concomitant ASMs was found between sleep quality groups, qualitative effects of specific agents on sleep architecture and neurocognitive functioning cannot be excluded.

Taken together, our results support a model in which poor sleep amplifies emotional dysregulation and executive impairment in pediatric IGEs. While the subjective nature of the sleep measures employed warrants interpretive caution, the consistency of associations across multiple validated instruments supports the plausibility of the proposed relationships and their clinical relevance.

Beyond its role as a physiological regulator, sleep quality may also act as a proxy for unmet emotional needs and internalizing traits. Clinically, poor sleep may serve as a sensitive early marker of hidden affective distress, highlighting the value of routine sleep screening in epilepsy care alongside targeted therapeutic strategies to improve sleep quality and hygiene. Neurobiologically, alterations in sleep architecture, particularly reduced restorative slow‐wave sleep, likely interact with limbic and prefrontal circuits, undermining emotion regulation and reinforcing internalizing symptomatology. Recognizing and addressing sleep impairment may therefore represent a critical avenue for improving not only seizure control but also the broader neuropsychiatric outcomes of children and adolescents living with IGEs.

Based on our findings, multiple research directions can be pursued to deepen the understanding of the intricate relationships among sleep, cognition, and emotional regulation in IGEs, as detailed in the future research agenda (as shown in Table 5).

**TABLE 5:** Future research agenda for the IGE population.

Focus area	Key research questions	Proposed methodologies	Expected impact
Objective Sleep Profiling	How do alterations in sleep architecture and circadian rhythms relate to seizure control, emotion, and executive function?	Polysomnography Actigraphy EEG spectral analysis Longitudinal sleep‐monitoring	Identify sleep biomarkers predictive of neuropsychiatric and cognitive outcomes
2 Neural Mechanism and Connectivity	Which brain circuits mediate the interaction between sleep, emotional regulation, and executive functioning?	Multimodal neuroimaging (fMRI, DTI, EEG–fMRI) Network analysis	Clarify fronto‐thalamo‐limbic dysconnectivity underlying IGE‐related dysfunction
3 Sleep‐Targeted Interventions	Can improving sleep quality enhance cognitive and emotional outcomes in IGE?	Randomized controlled trials on CBT‐I, melatonin, and chronotherapy	Establish sleep as a modifiable therapeutic target beyond seizure control
4 Clinical Translation and Implementation	How can sleep and emotional screening be incorporated into epilepsy management?	Multicenter validation PROMs Implementation studies for a sleep‐informed management of pediatric IGE	Inform evidence‐based, multidisciplinary care pathways

## AUTHOR CONTRIBUTIONS

**Carlo Alberto Quaranta:** Conceptualization, Methodology, investigations, data curation, writing—original draft preparation, writing—reviewing and editing. **Martina Paola Zanaboni:** Investigations, data curation, writing—reviewing and editing. **Elena Ripamonti:** Writing—reviewing and editing, investigation. **Gaia Garghetti:** Writing—reviewing and editing, investigation. **Massimiliano Celario:** Writing—reviewing and editing. **Serena Grumi:** Writing—reviewing and editing, investigation. **Helene Vitali:** Writing—reviewing and editing, investigation. **Valentina De Giorgis:** Conceptualization, methodology, writing—review and editing; **Ludovica Pasca:** Conceptualization, methodology, data curation, writing— original draft preparation, writing—reviewing and editing, supervision.

## CONFLICT OF INTEREST STATEMENT

Dr. M.P. Zanaboni has received consultancy fees from Vitaflo. Dr. V. De Giorgis has received research grants from Jazz Pharmaceuticals, speaker and consultancy fees from Nutricia Gmbh, Vitaflo, Dr. Schar Kanso, and has served as a consultant/advisor for Longboard Pharmaceuticals and Dr. Schar Kanso. The remaining authors have no conflict of interest to disclose.

**Test yourself**

1. In the study cohort, what percentage of adolescents with IGEs showed poor sleep quality on the PSQI (score >5), despite only 16.1% spontaneously reporting sleep problems?
  - 14.8%
  - 28.6%
  - 42.3%
  - 57.1%
2. Which executive function domains were significantly more impaired in poor sleepers (PSQI >5) according to self‐report BRIEF scores?
  - Inhibition, Planning, and Verbal Memory
  - Processing Speed, Attention, and BRI
  - Shift, Emotional Regulation, CRI, ERI, and GEC
  - Working Memory, Initiation, and Monitoring only
3. Why did significant associations between sleep quality and emotional problems emerge in self‐reports (YSR, SAFA) but not in parent‐proxy CBCL reports?
  - Parents completed the CBCL incorrectly
  - The CBCL is not validated for epilepsy populations
  - Sleep disturbances reflect internal subjective experiences that adolescents perceive and report better than caregivers
  - Parents systematically overestimate their child's wellbeing

*Answers may be found in the* supporting information.

## Supporting information


Table S1.

Data S1.

## Data Availability

The data that support the findings of this study are available from the corresponding author upon reasonable request.
