# Supplementary material for: The hidden burden of sleep in adolescents with idiopathic generalized epilepsy
Source: Epileptic Disord. 2026 Jul 11;28(5):1520–31. doi: 10.1002/epd2.70288 (PMC13695483; doi:10.1002/epd2.70288)
Supplement: Supplementary file 1 [file EPD2-28-1520-s001.docx]

**Supplementary Results Section:**

| **Domain** | **N** | **Mean** | **SD** | **% Moderately elevated**  **(60<T< 64)** | **% Potentially Elevated**  **(65<T<69)** | **% Clinically Elevated (T≥70)** |
| --- | --- | --- | --- | --- | --- | --- |
| **Parent Report** | | | | | | |
| Inhibition | 26 | 49,2 | 8,7 | 7,70% | 7,70% | 0% |
| Self-Monitoring | 26 | 48,1 | 9,5 | 3,80% | 3,80% | 3,80% |
| Shift | 26 | 52,5 | 11,9 | 11,40% | 11,50% | 3,80% |
| Emotional Control | 26 | 48,3 | 11,8 | 7,60% | 3,80% | 7,70% |
| Initiation | 26 | 49,7 | 9,8 | 7,60% | 0% | 3,80% |
| Working Memory | 26 | 57,8 | 13,8 | 15,30% | 11,50% | 15,40% |
| Planning/Organization | 26 | 53,7 | 13,7 | 11,50% | 0% | 19,20% |
| Task Monitoring | 26 | 54,8 | 14,9 | 7,60% | 11,50% | 19,20% |
| Organization of Materials | 26 | 48,6 | 7,8 | 7,60% | 3,80% | 0% |
| **Self-Report** | | | | | | |
| Inhibition | 28 | 51,2 | 10,9 | 10,80% | 14,30% | 3,60% |
| Self-Monitoring | 28 | 50,4 | 13,3 | 3,60% | 7,10% | 10,70% |
| Shift | 28 | 54 | 13,4 | 7,20% | 10,70% | 10,70% |
| Emotional Control | 28 | 48,9 | 10,4 | 7,20% | 7,10% | 3,60% |
| Task Completion | 28 | 53,1 | 12,1 | 10,70% | 7,10% | 7,10% |
| Working Memory | 28 | 53,3 | 10,3 | 3,60% | 3,60% | 7,10% |
| Planning/Organization | 28 | 50,9 | 11,1 | 17,80% | 3,60% | 3,60% |
|  | | | | | | |
| **Indexes** | | | | | | |
| BRI_parent | 26 | 48,04 | 9,6 | 3,8% | 3,8% | 3,8% |
| ERI_parent | 26 | 50,08 | 12,3 | 0% | 7,60% | 11,40% |
| CRI_parent | 26 | 52,7 | 12,8 | 11,50% | 11,40% | 11,40% |
| GEC_parent | 26 | 51,4 | 12,4 | 11,50% | 11,40% | 7,60% |
|  | | | | | | |
| BRI_self | 28 | 50,8 | 11,3 | 7,20% | 21,40% | 0% |
| ERI_self | 28 | 51,9 | 12,6 | 3,60% | 3,60% | 14,30% |
| CRI_self | 28 | 52,7 | 12,2 | 7,20% | 3,60% | 7,10% |
| GEC_self | 28 | 52,5 | 12,6 | 14,20% | 3,60% | 10,70% |

**Table 1**. EF scoring of our cohort (Parent and self-report). Abbreviations: N=number, SD= standard deviation, BRI= Behavioral Regulation, ERI= Emotional Regulation, CRI= Cognitive Regulation, GEC= Global executive score.

| **Source** | **CBCL** | | **N** | **Mean** | **SD** | **% Borderline** | **% Clinically relevant**  **( T ≥ 65)** |
| --- | --- | --- | --- | --- | --- | --- | --- |
| **Parent** | **Empirical Scales** | Internalizing problems | 27 | 56,85 | 12,47 | 12% | 20% |
|  |  | Externalizing problems | 26 | 48,73 | 8,93 | 0% | 0% |
|  |  | Total problems | 27 | 53,63 | 12,60 | 12% | 8% |
|  |  | Anxious/Depressed | 26 | 57,50 | 7,32 | 20% | 4% |
|  |  | Withdrawn/Depressed | 27 | 58,48 | 10,33 | 0% | 11,5% |
|  |  | Somatic complaints | 26 | 59,69 | 9,32 | 3,8% | 19,2% |
|  |  | Social problems | 27 | 56,41 | 6,76 | 8% | 4% |
|  |  | Thought problems | 26 | 55,77 | 5,67 | 12% | 0% |
|  |  | Attention problems | 27 | 59,48 | 9,46 | 15,4% | 11,5% |
|  |  | Oppositional behavior | 26 | 54,15 | 4,32 | 0% | 0% |
|  |  | Aggressive behavior | 26 | 53,08 | 4,20 | 4% | 0% |
|  | **DSM- oriented scales** | Depressive problems | 27 | 59,59 | 8,63 | 12% | 16% |
|  |  | Anxiety problems | 27 | 60,22 | 8,75 | 7,7% | 26,9% |
|  |  | Somatic problems | 27 | 57,85 | 8,44 | 3,8% | 11,5% |
|  |  | Attention deficit | 26 | 55,81 | 5,97 | 0% | 4% |
|  |  | Oppositional problems | 27 | 53,56 | 4,12 | 3,8% | 0% |
|  |  | Conduct problems | 27 | 53,44 | 4,48 | 0% | 0% |
|  |  | Cognitive slowing ASEBA | 27 | 59,33 | 8,16 | 15,4% | 15,4% |
|  |  | Obsessive-compulsive problems ASEBA | 27 | 55,74 | 6,05 | 11,5% | 0% |
|  |  | Stress problems ASEBA | 26 | 58,81 | 8,28 | 0% | 16% |
| **Patient** | **Empirical Scales** | Internalizing problems | 26 | 57,85 | 11,30 | 11,5% | 15,4% |
|  |  | Externalizing problems | 26 | 51,73 | 8,99 | 11,5% | 0% |
|  |  | Total problems | 26 | 54,38 | 10,67 | 19,2% | 3,8% |
|  |  | Anxious/Depressed | 26 | 60,00 | 9,63 | 15,4% | 19,2% |
|  |  | Withdrawn/Depressed | 26 | 60,19 | 10,84 | 19,2% | 15,4% |
|  |  | Somatic complaints | 25 | 55,72 | 8,49 | 4% | 4% |
|  |  | Social problems | 25 | 56,88 | 6,99 | 12% | 4% |
|  |  | Thought problems | 25 | 56,24 | 7,63 | 0% | 4% |
|  |  | Attention problems | 25 | 57,68 | 7,81 | 8% | 4% |
|  |  | Oppositional behavior | 26 | 54,19 | 4,26 | 0% | 0% |
|  |  | Aggressive behavior | 26 | 54,92 | 5,81 | 7,7% | 3,8% |
|  | **DSM- oriented scales** | Depressive problems | 26 | 58,46 | 8,67 | 15,4% | 11,5% |
|  |  | Anxiety problems | 26 | 59,23 | 7,87 | 19,2% | 7,7% |
|  |  | Somatic problems | 25 | 56,04 | 8,9 | 3,8% | 3,8% |
|  |  | Attention deficit | 25 | 55,84 | 6,06 | 8% | 4% |
|  |  | Oppositional problems | 25 | 54,56 | 5,25 | 4% | 4% |
|  |  | Conduct problems | 25 | 52,44 | 3,31 | 0% | 0% |
|  |  | Obsessive-compulsive problems ASEBA | 26 | 58,31 | 8,77 | 19,2% | 11,5% |
|  |  | Stress problems ASEBA | 25 | 59,32 | 9,81 | 11,5% | 19,2% |
|  |  | Positive qualities ASEBA | 26 | 47,38 | 8,91 | 3,8% | 0% |

**Table 2.** CBCL (Parent Report) and YSR (Youth Self Report) of our cohort. Note: T scores between 65 and 69 are considered borderline; T scores above 70 are considered clinically significant.

Note: CBCL= Child Behavior Checklist, YSR= Youth Self-Report; N = number of participants; SD = standard deviation.

| **SAFA-Scale** | **N** | **Mean** | **SD** | **% At Risk** | **% Clinically relevant**  **(T>65)** |
| --- | --- | --- | --- | --- | --- |
| **SAFA-ANXIETY** | | | | | |
| Anxiety Total Score | 28 | 52,79 | 11,874 | 14,3 | 7,1 |
| Generalized Anxiety | 28 | 53,93 | 13,862 | 14,3 | 17,9 |
| Social Anxiety | 28 | 58 | 10,801 | 10,7 | 10,7 |
| Separation Anxiety | 28 | 48,5 | 12,509 | 3,6 | 7,1 |
| School Anxiety | 28 | 48,18 | 9,031 | 3,6 | 0 |
| **SAFA-DEPRESSION** | | | | | |
| Depression Total Score | 28 | 52,18 | 12,472 | 3,6 | 14,3 |
| Depressed Mood | 28 | 49,86 | 10,498 | 3,6 | 7,1 |
| Anhedonia-Disinterest | 28 | 50,61 | 9,287 | 3,6 | 7,1 |
| Irritable Mood | 28 | 51,68 | 13,603 | 7,1 | 10,7 |
| Inadequacy-Low Self-Esteem | 28 | 54,5 | 14,125 | 3,6 | 21,4 |
| Insecurity | 28 | 53,11 | 10,436 | 3,6 | 10,7 |
| Guilt | 28 | 47,32 | 11,595 | 0 | 7,1 |
| Hopelessness | 28 | 54,11 | 11,393 | 10,7 | 10,7 |
| Simulation | 28 | 58,18 | 10,801 | 10,7 | 14,3 |
| **SAFA-SOMATIC SYMPTOMS AND HYPOCONDRIA** | | | | | |
| Somatic Symptoms and Hypochondria Total Score | 28 | 47,89 | 9,7 | 7,1 | 3,6 |
| Somatic Symptoms | 28 | 48,43 | 10,075 | 3,6 | 3,6 |
| Hypochondria | 28 | 46,39 | 8,937 | 0 | 3,6 |

**Table 3.** SAFA Assessments of our cohort. Note: T scores between 65 and 69 are considered as pathology risk; T scores above 70 are considered index of pathology.

Note: SAFA=Self-administered Psychiatric Scales for children and adolescents; N=number of participants; SD = standard deviation.

| **Dream Variable** | **N (%)** |
| --- | --- |
| Dream recall - Never/rarely | 11 (44%) |
| Dream recall - Once or more per week | 14 (56%) |
| Awakenings due to nightmares - Never/rarely | 20 (80%) |
| Awakenings due to nightmares - Sometimes/often/very often | 5 (20%) |
| Difficulty falling asleep due to nightmares - Never/rarely | 19 (76%) |
| Difficulty falling asleep due to nightmares - Sometimes/often/very often | 6 (24%) |
| Fear due to nightmares - Never/rarely | 21 (84%) |
| Fear due to nightmares - Sometimes/often/very often | 4 (16%) |
| Difficulty sleeping due to nightmares - Never/rarely | 20 (80%) |
| Difficulty sleeping due to nightmares - Sometimes/often/very often | 5 (20%) |
| Shortness of breath due to nightmares - Never/rarely | 22 (84%) |
| Shortness of breath due to nightmares - Sometimes/often/very often | 4 (16%) |
| Dizziness due to nightmares - Never/rarely | 23 (92%) |
| Dizziness due to nightmares - Sometimes/often/very often | 2 (8%) |
| Exhaustion due to nightmares - Never/rarely | 20 (80%) |
| Exhaustion due to nightmares - Sometimes/often/very often | 5 (20%) |
| Palpitations due to nightmares - Never/rarely | 21 (84%) |
| Palpitations due to nightmares - Sometimes/often/very often | 4 (16%) |
| Sweating due to nightmares - Never/rarely | 21 (84%) |
| Sweating due to nightmares - Sometimes/often/very often | 4 (16%) |
| Chills due to nightmares - Never/rarely | 23 (92%) |
| Chills due to nightmares - Sometimes/often/very often | 2 (8%) |
| Nausea due to nightmares - Never/rarely | 21 (84%) |
| Nausea due to nightmares - Sometimes/often/very often | 4 (16%) |
| Stomach ache due to nightmares - Never/rarely | 23 (92%) |
| Stomach ache due to nightmares - Sometimes/often/very often | 2 (8%) |
| Chest tightness due to nightmares - Never/rarely | 21 (84%) |
| Chest tightness due to nightmares - Sometimes/often/very often | 4 (16%) |
| Dry mouth due to nightmares - Never/rarely | 21 (84%) |
| Dry mouth due to nightmares - Sometimes/often/very often | 4 (16%) |
| Fear of dying due to nightmares - Never/rarely | 20 (80%) |
| Fear of dying due to nightmares - Sometimes/often/very often | 5 (20%) |
| Sore throat due to nightmares - Never/rarely | 24 (96%) |
| Sore throat due to nightmares - Sometimes/often/very often | 1 (4%) |
| Ability to recall dreams after nightmares - Never/rarely | 18 (72%) |
| Ability to recall dreams after nightmares - Sometimes/often/very often | 7 (28%) |
| Daytime sleepiness due to nightmares - Never/rarely | 19 (76%) |
| Daytime sleepiness due to nightmares - Sometimes/often/very often | 6 (24%) |
| Irritability/anxiety due to nightmares - Never/rarely | 17 (68%) |
| Irritability/anxiety due to nightmares - Sometimes/often/very often | 8 (32%) |
| Difficulty in routine due to nightmares - Never/rarely | 23 (92%) |
| Difficulty in routine due to nightmares - Sometimes/often/very often | 2 (8%) |
| Difficulties in relationships due to nightmares - Never/rarely | 23 (92%) |
| Difficulties in relationships due to nightmares - Sometimes/often/very often | 2 (8%) |
| Difficulties in activities due to nightmares - Never/rarely | 22 (88%) |
| Difficulties in activities due to nightmares - Sometimes/often/very often | 3 (12%) |
| Mood disorders due to nightmares - Never/rarely | 19 (76%) |
| Mood disorders due to nightmares - Sometimes/often/very often | 6 (24%) |
| Memory difficulties due to nightmares - Never/rarely | 22 (88%) |
| Memory difficulties due to nightmares - Sometimes/often/very often | 3 (12%) |
| Visual sensory content in dreams - No | 6 (24%) |
| Visual sensory content in dreams - Yes | 19 (76%) |
| Auditory sensory content in dreams - No | 4 (16%) |
| Auditory sensory content in dreams - Yes | 21 (84%) |
| Tactile sensory content in dreams - No | 9 (36%) |
| Tactile sensory content in dreams - Yes | 16 (64%) |
| Gustatory sensory content in dreams - No | 21 (84%) |
| Gustatory sensory content in dreams - Yes | 4 (16%) |
| Olfactory sensory content in dreams - No | 21 (84%) |
| Olfactory sensory content in dreams - Yes | 4 (16%) |
| Dream recall frequency - Almost every morning / several times a week / once a week | 9 (36%) |
| Dream recall frequency - Never / up to 3 times per month | 16 (64%) |
| Emotional intensity of dreams - Not intense | 12 (48%) |
| Emotional intensity of dreams - Intense | 13 (52%) |
| Emotional tone of dreams - Negative | 5 (17.9%) |
| Emotional tone of dreams - Neutral | 13 (46.4%) |
| Emotional tone of dreams - Positive | 6 (21.4%) |
| Nightmare frequency - Several times a week/month | 10 (40%) |
| Nightmare frequency - Never / max 2-3 times per year | 15 (60%) |
| Nightmare distress - Absent | 14 (58.3%) |
| Nightmare distress - Present | 10 (41.7%) |
| Recurrent nightmares - No | 17 (70.8%) |
| Recurrent nightmares - Yes | 7 (29.2%) |
| Percentage of recurrent nightmares <50% | 15 (68.2%) |
| Percentage of recurrent nightmares ≥50% | 7 (31.8%) |
| Nightmares in childhood - Several times a week/month | 6 (26.1%) |
| Nightmares in childhood - Never / max 2-3 times per year | 17 (73.9%) |
| Lucid dreams - Several times a week/month | 9 (36%) |
| Lucid dreams - Never / max 2-3 times per year | 16 (64%) |
| Meaning attributed to dreams - Low | 13 (52%) |
| Meaning attributed to dreams - High | 12 (48%) |
| Interest in dreams - Low | 13 (52%) |
| Interest in dreams - High | 12 (48%) |
| Desire to know more about dreams - Low | 8 (32%) |
| Desire to know more about dreams - High | 17 (68%) |
| Life enrichment from dream recall/interpretation - Low | 12 (48%) |
| Life enrichment from dream recall/interpretation - High | 13 (52%) |
| Dreaming is interesting - No | 3 (12%) |
| Dreaming is interesting - Yes | 22 (88%) |
| Reflection on dreams provides self-knowledge - No | 9 (36%) |
| Reflection on dreams provides self-knowledge - Yes | 16 (64%) |
| Impulses and goals - No | 13 (52%) |
| Impulses and goals - Yes | 12 (48%) |
| Frequency of dream sharing - Several times a week/month | 8 (32%) |
| Frequency of dream sharing - Never / up to 3 times per year | 17 (68%) |
| Recording and transcription - Several times a week/month | 0 (0%) |
| Recording and transcription - Never / up to 3 times per year | 25 (100%) |
| Influence on mood - Several times a week/month | 8 (32%) |
| Influence on mood - Never / up to 3 times per year | 17 (68%) |
| Creative ideas - Several times a week/month | 8 (32%) |
| Creative ideas - Never / up to 3 times per year | 17 (68%) |
| Problem solving - Several times a week/month | 5 (20%) |
| Problem solving - Never / up to 3 times per year | 20 (80%) |
| Déjà-vu - Several times a week/month | 1 (44%) |
| Déjà-vu - Never / up to 3 times per year | 14 (56%) |
| Reading about dreams - No | 16 (64%) |
| Reading about dreams - Yes | 9 (36%) |
| Help from literature - No | 2 (25%) |
| Help from literature - Yes | 6 (75%) |

**Table 4.** Dream Assessment of our cohort.

| **PSQI Correlation Coefficient** | | |
| --- | --- | --- |
|  | **R pearson** | **P-value**  **(*Significance FDR)** |
| **BRIEF self-report** | | |
| Self-monitoring | 0.491 ** | .008* |
| Shift | 0.620** | .000* |
| Emotion regulation | 0.573 ** | .001* |
| Task completion | 0.582 ** | .001* |
| Working memory | 0.560 * | .002* |
| Planning/organization | 0.490 * | .008* |
| BRI | 0.399 * | .036* |
| ERI | 0.657 ** | .000* |
| CRI | 0.578** | .001* |
| GEC | 0.609 ** | .001* |
| **SAFA** | | |
| Anxiety | 0.533 ** | .003* |
| Generalized anxiety | 0.477 * | .010* |
| Social anxiety | 0.428 * | .023* |
| Separation anxiety | 0.300 | .121 |
| School-related anxiety | 0.512 ** | .005* |
| Depression | 0.452 * | .016* |
| Depressed mood | 0.335 | .081 |
| Anhedonia/lack of interest | 0.376 * | .048 |
| Irritable mood | .341 | .076 |
| Feelings of inadequacy, low self-esteem | 0.469 * | .012* |
| Emotional Insecurity | 0.275 | .157 |
| Sense of guilt | 0.296 | .126 |
| Despair | 0.469 ** | .012* |
| Simulation | -0.481 ** | .010* |
| Somatic Symptoms and Hypochondria | 0.842 ** | .000* |
| Somatic symptoms | 0.832 ** | .000* |
| Hypochondria | 0.588 ** | .001* |
| **CBCL** | | |
| Internalizing problems | 0.411 * | .041 |
| Externalizing problems | 0.343 | .093 |
| Total problems | 0.409 * | .042 |
| Anxious/depressive | 0.360 | .077 |
| Withdrawn/ depressed | 0.267 | .188 |
| Somatic complaints | 0.417 * | .034 |
| Social problems | 0.283 | .170 |
| Thought problems | 0.469 * | .018 |
| Attention problems | 0.500 ** | .009 |
| Oppositional behaviour | 0.481 * | .013 |
| Aggressive behaviour | 0.241 | .245 |
| Depressive problems | 0.461 * | .020 |
| Anxiety problems | 0.252 | .214 |
| Somatic problems | 0.377 | .058 |
| Attention deficit | 0.461 * | .020 |
| Oppositional behavior | 0.154 | .453 |
| Conduct problems | 0.409 * | .038 |
| Cognitive slowing (ASEBA) | .285 | .159 |
| Obsessive-compulsive problems (ASEBA) | 0.559 ** | .003 |
| Stress problems (ASEBA) | 0.472 * | .017 |
| **YSR** | | |
| Internalizing problems | 0.667 ** | .000* |
| Externalizing problems | 0.529 ** | .005* |
| Total problems | 0.689 ** | .000* |
| Anxious/depressed | 0.636 ** | .000* |
| Withdrawn/depressed | 0.363 | .068 |
| Somatic complaints | 0.737 ** | .000* |
| Social problems | 0.483 * | .014* |
| Thought problems | 0.663 ** | .000* |
| Attention problems | 0.541 ** | .005* |
| Oppositional behaviour | 0.262 | .195 |
| Aggressive behaviour | 0.567 ** | .003* |
| Depressive problems | 0.690 ** | .000* |
| Anxiety problems | 0.573 ** | .002* |
| Somatic problems | 0.585 ** | .002* |
| Attention deficit | 0.394 | .051 |
| Oppositional problems | 0.710 ** | .000* |
| Conduct problems | .299 | .139 |
| Obsessive-compulsive problems (ASEBA) | 0.535 ** | .005* |
| Stress problems (ASEBA) | 0.636  ** | .000* |
| Positive qualities ( ASEBA) | 0.055 | .790 |

**Note:** *p<.05; **p<.01.Abbreviations: BRIEF=Behaviour Rating Inventory of Executive Function; SAFA=Self-administered Psychiatric Scales for children and adolescents; CBCL= Child Behavior Checklist, YSR= Youth Self-Report.
