# Supplementary material for: The hidden burden of sleep in adolescents with idiopathic generalized epilepsy
Source: Epileptic Disord. 2026 Jul 11;28(5):1520–31. doi: 10.1002/epd2.70288 (PMC13695483; doi:10.1002/epd2.70288)
Supplement: Supplementary file 2 [file EPD2-28-1520-s002.docx]

**Test yourself**

**Answers:**

1-B; 2-C; 3-C
